# Depletion of CPNE7 sensitizes colorectal cancer to 5‐fluorouracil by downregulating ATG9B expression

**DOI:** 10.1111/jcmm.18261

**Published:** 2024-03-25

**Authors:** Weile Xu, Yujie Tang, Yang Yang, Changjing Wang, Chen Liu, Jianqing Zhang, Lianmei Zhao, Guiying Wang

**Affiliations:** ^1^ The Department of General surgery The Second Hospital of Hebei Medical University Shijiazhuang Hebei China; ^2^ The Department of General surgery Hebei Chest Hospital Shijiazhuang Hebei China; ^3^ The Second Department of Surgery The Fourth Hospital of Hebei Medical University Shijiazhuang Hebei China; ^4^ The Department of Gastrointestinal surgery The Third Hospital of Hebei Medical University Shijiazhuang Hebei China; ^5^ Scientific Research Center The Fourth Hospital of Hebei Medical University Shijiazhuang Hebei China

**Keywords:** ATG9B, cell proliferation, chemosensitivity, colorectal cancer, CPNE7

## Abstract

We aimed to explore the biological function of CPNE7 and determine the impact of CPNE7 on chemotherapy resistance in colorectal cancer (CRC) patients. According to the Gene Expression Profiling Interactive Analysis database and previously published data, CPNE7 was identified as a potential oncogene in CRC. RT‐qPCR and Western blotting were performed to verify the expression of CPNE7. Chi‐square test was used to evaluate the associations between CPNE7 and clinical features. Cell proliferation, colony formation, cell migration and invasion, cell cycle and apoptosis were assessed to determine the effects of CPNE7. Transcriptome sequencing was used to identify potential downstream regulatory genes, and gene set enrichment analysis was performed to investigate downstream pathways. The effect of CPNE7 on 5‐fluorouracil chemosensitivity was verified by half maximal inhibitory concentration (IC50). Subcutaneous tumorigenesis assay was used to examine the role of CPNE7 in sensitivity of CRC to chemotherapy in vivo. Transmission electron microscopy was used to detect autophagosomes. CPNE7 was highly expressed in CRC tissues, and its expression was correlated with T stage and tumour site. Knockdown of CPNE7 inhibited the proliferation and colony formation of CRC cells and promoted apoptosis. Knockdown of CPNE7 suppressed the expression of ATG9B and enhanced the sensitivity of CRC cells to 5‐fluorouracil in vitro and in vivo. Knockdown of CPNE7 reversed the induction of the autophagy pathway by rapamycin and reduced the number of autophagosomes. Depletion of CPNE7 attenuated the malignant proliferation of CRC cells and enhanced the chemosensitivity of CRC cells to 5‐fluorouracil.

## 1 INTRODUCTION

Colorectal cancer (CRC) has the fourth and second highest incidence and mortality rates worldwide, respectively, and it causes approximately one million deaths per year.^1^, ^2^ CRC is the second most common cancer, and approximately 4,00,000 new cases are diagnosed every year in China.^3^, ^4^ Most CRC cases are already in the middle‐late stage at diagnosis, and the 5‐year survival rate is only approximately 30%.^5^, ^6^ As an increasing number of tumour biomarkers and therapeutic targets are discovered and new drugs, such as bevacizumab and cetuximab, are being developed, the prognosis of patients is improving.^7^, ^8^ However, only 40% of patients benefit from these drugs.^9^ Therefore, new molecular mechanisms underlying CRC pathogenesis need to be elucidated to reveal new tumour biomarkers and new therapeutic targets.

The treatment of CRC mainly includes preoperative neoadjuvant radiotherapy, surgical treatment and postoperative chemotherapy.^5^ Chemotherapy regimens with 5‐fluorouracil as the main agent are the main treatment strategy.^10^, ^11^ However, chemotherapy is not always effective because many patients exhibit innate or acquired resistance to drugs.^12^ Less than 50% of patients benefit from preoperative neoadjuvant therapy.^13^ Some patients even experience tumour progression during preoperative neoadjuvant therapy, which makes them ineligible for curative surgery.^14^ During postoperative chemotherapy, many patients also develop chemoresistance, which leads to recurrence and metastasis, thus seriously affecting the survival.^15^, ^16^ Therefore, it is crucial to actively search for new mechanisms underlying chemoresistance.

Copine‐7 (CPNE7) is a dental epithelium‐derived factor that has been extensively studied in the oral cavity; it can affect cell proliferation, migration and differentiation^17^, ^18^ and is associated with neurotransmitters and sleep.^19^ Recent studies have demonstrated that CPNE7 can affect the metastasis of oral cancer cells^20^ and induce autophagy.^21^ Autophagy plays an important role in chemotherapy resistance in cancers such as CRC,^22^ gastric cancer^23^ and other cancers.^24^ However, the functions of CPNE7 in CRC and whether CPNE7 affects autophagy and thus chemoresistance are unclear.

Here, we report that CPNE7 expression is upregulated in CRC. We also verified by in vitro and in vivo experiments that CPNE7 can promote cell proliferation and chemoresistance in CRC. In addition, our further study confirmed that CPNE7 can affect the expression of the autophagy‐related gene ATG9B. In conclusion, this study revealed that CPNE7 can function as an oncogene in CRC and affect the sensitivity of CRC to 5‐fluorouracil. This study identified a new potential therapeutic target and provided a theoretical basis for future antitumor drug development.

## 2 MATERIALS AND METHODS

### 2.1 Tumour specimens

Eighty‐five fresh CRC specimens and paired normal specimens were frozen in liquid nitrogen immediately after surgical resection at the Fourth Hospital of Hebei Medical University from 2020 to 2021, and these specimens were subjected to real‐time quantitative PCR(RT‐qPCR) and Western blotting. The specimens were collected from patients who met the following conditions: (1) Patients were diagnosed with colorectal adenocarcinoma and did not receive preoperative neoadjuvant therapy; (2) Tumour tissue was obtained from the non‐necrotic area of the tumour, and paired normal tissues were obtained from the upper and lower margins of the tumour at a distance of approximately 15 cm. The detailed information about the patients, such as sex and age, is shown in Table S1. The study protocol was approved by the Ethics Committee of the Fourth Hospital of Hebei Medical University, and all the patients signed an informed consent forms.

### 2.2 Cell culture

The human CRC cell lines used for the experiments were provided by the Research Center of the Fourth Hospital of Hebei Medical University. All cell lines were authenticated by short tandem repeat (STR) profiling, and mycoplasma contamination was excluded. The HCT116 and HT29 cell lines were maintained in RPMI‐1640 medium supplemented with 10% foetal bovine serum (FBS). SW480, SW620, LOVO, RKO and NCM460 cells were maintained in Dulbecco's modified Eagle's medium (DMEM) supplemented with 10% foetal bovine serum (FBS). All the cell lines were cultured at 37°C in 5% CO_2_.

### 2.3 RNA extraction and RT‐qPCR

Total RNA was extracted using the RNA simple Total RNA Kit (DP419, TIANGEN BIOTECH BEIJING CO., Ltd.). After checking the purity (1.8 < OD260/OD280 < 2.0) and concentration (1000–1500 ng/μL), total RNA was reverse transcribed into cDNA by using a Reverse Transcription System (Promega, Wisconsin, USA) according to the manufacturer's instructions. Then, the cDNA was subjected to quantitative real‐time PCR (RT‐qPCR) analysis on a 7500 RT‐qPCR System (Applied Biosystems, USA) with qPCR Mix (Promega, Wisconsin, USA). Using the SYBR Green method, amplification was completed under the following reaction conditions: 95°C for 2 min; denaturation at 95°C for 15 s; annealing and extension at 60°C for 1 min; and 40 cycles of melting at 95°C for 15 s, 60°C for 1 min, and 95°C for 15 s. All samples were run in triplicate, and the results were analysed with the 2^−ΔΔCt^ method.

The primers used in this study are listed as follows:

**Table:** 

CPNE7	Forward, 5′‐GCAGAACCGAGGTGGTCC‐3′ Reverse, 5′‐TGCGTGTCGTACACCTCAAA‐3′
ATG9B	Forward, 5′‐CAGGCACCAGGAAGCCAGAA‐3′ Reverse, 5′‐GCAGGAAACAAAGTCCACAAAGC‐3′
GAPDH	Forward, 5′‐ GGACCTGACCTGCCGTCTAG‐3′ Reverse, 5′‐ GTAGCCCAGGATGCCCTTGA‐3′

### 2.4 Western blotting

Proteins were extracted from tissues of patients with colorectal adenocarcinoma from CRC cell lines and subjected to Western blot analysis. A BCA protein assay kit (Beijing Solarbio Science&Technology Co., Ltd) was used to measure the protein concentrations according to the manufacturer's instructions. Forty micrograms of total protein was subject to SDS‐PAGE and then transferred to polyvinylidene difluoride (PVDF) membranes. After blocking with Tris buffered saline (TBS) supplemented with 1% Tween‐20 and 5% skim milk for 1 h at room temperature, the membranes were probed with primary antibodies including anti‐ATG9B (25131, Signalway Antibody, USA), anti‐CPNE7 (ab68547, Abcam, USA), anti‐LC3 I/II (PM036, MBL, Japan), anti‐p62 (PM045, MBL, Japan) anti‐GAPDH (AP0063, Bioworld, China) antibodies, at 4°C overnight. Subsequently, the membranes were washed three times with TBST for 10 min each, incubated with conjugated secondary antibodies for 1 h at room temperature, and washed three washes with TBST for 10 min each. The blots were visualized by an LI‐COR Odyssey 9120 Infrared Imaging System (LI‐COR Biosciences). We used Western blotting analysis to detect changes in the expression of the p62 and LC3‐I/II proteins in CPNE7‐knockdown HCT116 cells treated with 100 nmol/L rapamycin (Cayman Chemical, USA). Finally, the grayscale values of the target protein and GAPDH were detected by ImageJ software, and the relative expression level of the target protein was calculated as the grayscale value of the target protein/the grayscale value of GAPDH.

### 2.5 Transfection

The three separate siRNAs targeting CPNE7 mRNA and the corresponding negative control RNA were designed and synthesized by GenePharma and subsequently utilized in the transfection assay according to the manufacturer's instructions. The sequences of these siRNAs and the negative control RNA were as follows:

**Table:** 

si‐CPNE7‐1	5′‐CCATGACTTTGCCATCAATTT‐3′
si‐CPNE7‐2	5′‐GGAGGCCUUCAAAGUCUCUTT‐3′
si‐CPNE7‐3	5′‐CCCAAATACAAGCAGAAGAGA‐3′
si‐CPNE7‐NC	5′‐UUCUCCGAACGUGUCACGUTT‐3′

### 2.6 Lentiviral infection

Lentiviral particles were used to knock down CPNE7 at the mRNA level; these particles were packaged and concentrated by Genechem Co., Ltd. (shanghai, China). The sequence information of these shRNAs and negative control RNA was consistent with that of the siRNAs and negative control RNA. The cell infection assay was performed exactly as specified in the manufacturer's manual. After 72 h of infection, CPNE7‐knockdown cells were selected with 2 μg/mL puromycin until non‐resistant cells died. RT‐qPCR and Western blotting were both performed to determine the expression level of CPNE7.

### 2.7 MTS assay

The tetrazolium‐based MTS assay was used to determine the cell proliferation rate of CRC cells. The cells were divided into three groups (si‐NC, si‐1 and si‐3). The experimental procedure was performed according to the manufacturer's instructions (G3582, Promega, USA). Briefly, the trypsin‐digested cells were counted and then seeded into a 96‐well plate at a density of 1000 cells per well. Six parallel wells were established for each sample. After the cells had fully adhered to the bottom of the wells, detection was performed at 24, 48, 72 and 96 h. Before detection, 20 μL of MTS solution was added to each well. After incubating for 2 h, the absorbance at 492 nm (OD value) was detected on a microplate reader (BioTek). Data from three different experiments were collected and statistically analysed.

### 2.8 Determination of the half maximal inhibitory concentration (IC50)

A total of 10,000 cells were seeded into the wells of a 96‐well plate. After 24 h of incubation, the medium was replaced with complete fresh medium supplemented with gradient concentrations of 5‐fluorouracil. After another 48 h of incubation, an MTS assay kit was used to characterize cell viability according to the manufacturer's instructions. Five parallel replicate wells were included for each group. The IC50 values were calculated with GraphPad Prism 9.

### 2.9 Colony formation assay

The cells were divided into three groups (si‐NC, si‐1 and si‐3). Cells in the logarithmic growth phase of growth were digested, collected and counted. A total of 1000 cells were seeded into one well of six‐well plates, and incubated at 37°C in humidified air with 5% CO_2_. Each sample was analysed three times simultaneously. After 7 days of incubation, the colonies were gently washed with PBS, fixed in methanol for 10 min, and then stained with 2% crystal violet for 10 min. Next, the six‐well plates were gently rinsed with pure water to completely remove the crystalline violet solution and allowed to dry naturally. The colony number in each well was counted and statistically analysed.

### 2.10 Migration and invasion assays

The cells were divided into three groups (si‐NC, si‐1 and si‐3). As described, migration and invasion assays were performed with 6.5 mm Transwell (8.0 μm pore polyester membrane inserts) (Corning, Inc., NY, USA). For the invasion assay, the polyester membranes in the upper chambers were precoated with 100 μL of a 2% Matrigel solution (BD, New Jersey, USA). After digestion and collection, the cells were resuspended in serum‐free medium and diluted to a density of 2 million cells/mL. One hundred microlitres of each cell suspension was added into the upper chambers, which had been previously filled with 50 μL of serum‐free medium, and the lower chambers were subsequently filled with 700 μL of medium supplemented with 20% foetal bovine serum. After 16 h of incubation, the inserts were fixed with methanol for 10 min, stained with 2% crystal violet for 10 min, and then washed and dried. The cells were observed under a microscope and counted in five fields. All the experiments were repeated three times.

### 2.11 Cell cycle and apoptosis assays

The cells (si‐NC, si‐1 and si‐3) that were used for this experiment were digested, centrifuged and washed. After following the instructions of the cell cycle assay kit (MultiSciences Biotech, Hangzhou, China) and apoptosis assay kit (Elabscience Biotechnology, Wuhan, China), the cells were pended for flow cytometric analysis. For the cell cycle assay, 1.0 × 10^6^ cells were harvested and suspended in 1 mL of DNA staining solution and 10 μL of permeabilization solution. Next, the cells were incubated at room temperature in the dark for 30 min and subsequently measured. For the cell apoptosis assay, 5.0 × 10^5^ cells were digested, centrifuged, washed and resuspended in 500 μL of binding buffer. After 5 μL of Annexin V‐APC Reagent and 5 μL of 7‐AAD Reagent were added, the cell suspension was incubated at room temperature in the dark for 15–20 min and then analysed. All the experiments were repeated three times.

### 2.12 Transmission electron microscopy (TEM)

We collected HCT116 cells transfected with sh‐CPNE7‐1, sh‐CPNE7‐3 or sh‐CPNE7‐NC, preprocessed them as described in a previously published article,^25^ and then observed autophagosomes via transmission electron microscopy at the Electron Microscopy Center of Hebei Medical University.

### 2.13 In vivo xenograft assay

Twenty‐eight male BALB/c nude mice (4 weeks old) were purchased from SPF (Beijing) Biotechnology Co., Ltd, and housed under specific‐pathogen‐free (SPF) conditions for a week‐long adaptation period. The mice were subsequently randomized into four groups with seven mice in each group. In two groups, each group was subcutaneously injected with a total of 1.0 × 10^7^ sh‐CPNE7 cells, and the other two groups were subcutaneously injected with the same number of sh‐NC cells. When the tumours reached 1 mm^3^, the mice were treated with 5‐fluorouracil every 3 days. At the termination of 5‐fluorouracil intervention, the mice were sacrificed, and the subcutaneous tumours were extracted and weighed. Tumour volume was calculated using the following formula: (length×width^2^)/2. Random allocation, treatment, measurement and statistical analysis were completed by different researchers. All animal researchers have undergone training and assessment, and hold the certificate of laboratory animal practitioner. All the animal care and procedures were performed in accordance with national and institutional policies for animal health and well‐being and approved by Ethics Committee of the Fourth Hospital of Hebei Medical University.

### 2.14 Statistical analysis

Statistical analysis and bioinformatics analysis were conducted as previously described.^26^, ^27^ Specifically, Spearman correlation was used to evaluate the correlations between genes. For enumeration data, the chi‐square test was used when the theoretical frequency was greater than 5, while fisher exact probability method was used when the theoretical frequency was less than or equal to 5. For quantitative data, Student's *t*‐test was applied. Paired or unpaired *t*‐tests were used for comparisons of two groups. For comparisons of three of more groups, one‐way ANOVA was used. First, a normality test was performed. When the data were normally distributed, ANOVA was used. When the data did not conform to a normal distribution, the nonparametric test was used. Adjusted *p* values were used. For the xenograft assay, one‐way ANOVA was performed. *p* < 0.05 was considered to indicate statistical significance. All the statistical analyses were performed using GraphPad Prism 9, and all the experiments were performed three times. *p < 0.05, **p < 0.01, ***p < 0.001.

## 3 RESULTS

### 3.1 CPNE7 is highly expressed in CRC and is correlated with advanced T stage

**FIGURE 1:**
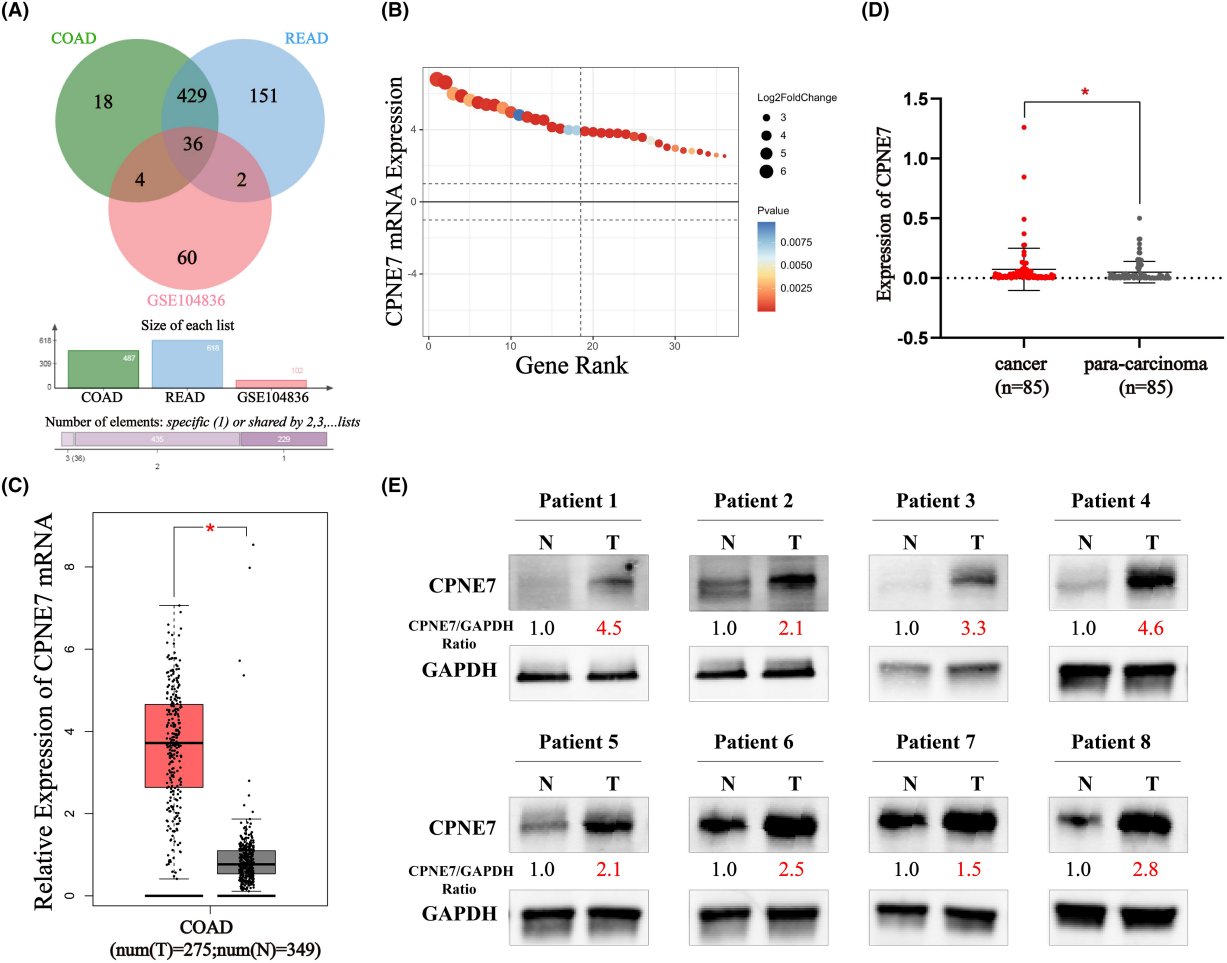
CPNE7 is highly expressed in CRC. (A) Venn Diagram of differentially expressed genes in the three databases (COAD from GEPIA, READ from GEPIA and GSE104836) (log_2_FC >2.5, *q* < 0.01). (B) 36 genes from three gene sets. (C) CPNE7 expression in CRC samples from the GEPIA database. (D) RT‐qPCR analysis of CPNE7 mRNA expression in 85 CRC tissues versus normal tissues. (E) Western blotting analysis of CPNE7 in eight patients with CRC. *p < 0.05.

As in our previous study (GSE104836), 102 genes were upregulated in CRC (log_2_FC >2.5, *q* < 0.01) (Table S2). Generally, highly expressed genes potentially have oncogenic functions. Therefore, we retrieved a collection of highly expressed genes (log_2_FC >2.5, *q* < 0.01) from the Gene Expression Profiling Interactive Analysis (GEPIA) database,^28^ of which 487 genes (Table S2) were highly expressed in COAD and 618 genes (Table S2) were highly expressed in READ. In addition, we found 36 genes that were present in all three gene sets (Figure 1A, Table S2). Among these 36 genes, we focused on CPNE7 as the main object of this study (Figure 1B). Previous studies have reported that CPNE7 functions as an oncogene in oral cancer and that its high expression can induce autophagy.^20^, ^21^ However, the role of CPNE7 in CRC is unclear. Given the high expression of CPNE7 found in public transcriptome sequencing data (Figure 1C), we suggest that it may have a cancer‐promoting effect. Accordingly, we subsequently examined the expression levels of CPNE7 in eight pairs of CRC tissues and normal tissues. Consistent with the RNA‐seq data, the protein level of CPNE7 was greater in CRC tissues than in paired normal tissues (Figure 1D, E). Finally, we evaluated the correlation between the CPNE7 expression level and the clinical characteristics of CRC patients. We found that high expression of CPNE7 was associated with advanced T stage and with particular tumour sites (Table 1). Collectively, these results revealed that CPNE7 may have a potential oncogenic function in CRC and could serve as a potential prognostic marker for CRC.

**TABLE 1:** Relationship between CPNE7 expression and clinicopathological characteristics in 85 CRC patients.

Clinicopathologic features	*n*	CPNE7		χ^2^	*p*‐Value
		Low [*n* (%)]	High [*n* (%)]		
All	85	42 (49.41%)	43 (50.59%)		
Age					
≤ 60	37	17 (45.95%)	20 (54.05%)	0.315	0.663
>60	48	25 (52.08%)	23 (47.92%)		
Gender					
Male	36	19 (52.78%)	17 (47.22%)	0.283	0.663
Female	49	23 (46.94%)	26 (53.06%)		
Tumour site					
Left	45	16 (35.56%)	29 (64.44%)	7.345	0.009*
Right	40	26 (65.00%)	14 (35.00%)		
Tumour size					
<5 cm	48	23 (47.92%)	25 (52.08%)	0.099	0.828
≥5 cm	37	19 (51.35%)	18 (48.65%)		
T stage					
T1 + T2	9	8 (88.89%)	1 (11.11%)	6.276	0.015*
T3 + T4	76	34 (44.74%)	42 (55.26%)		
N stage					
N0	45	23 (51.11%)	22 (48.89%)	0.110	0.829
N1 + 2	40	19 (47.50%)	21 (52.50%)		
TNM stage					
I + II	46	25 (54.35%)	21 (45.65%)	0.977	0.386
III + IV	39	17 (43.59%)	22 (56.41%)		
Tumour embolus					
Negative	74	36 (48.65%)	38 (51.35%)	0.133	0.757
Positive	11	6 (54.55%)	5 (45.45%)		
Nerve invasion					
Negative	68	32 (47.06%)	36 (52.94%)	0.753	0.427
Positive	17	10 (58.82%)	7 (41.18%)		
Pathological differentiation					
Well	19	8 (42.11%)	11 (57.89%)	1.018	0.601
Moderate	18	8 (44.44%)	10 (55.56%)		
Poor	48	26 (54.17%)	22 (45.83%)		

* *p* < 0.05.

### 3.2 High expression of CPNE7 inhibits apoptosis in CRC

**FIGURE 2:**
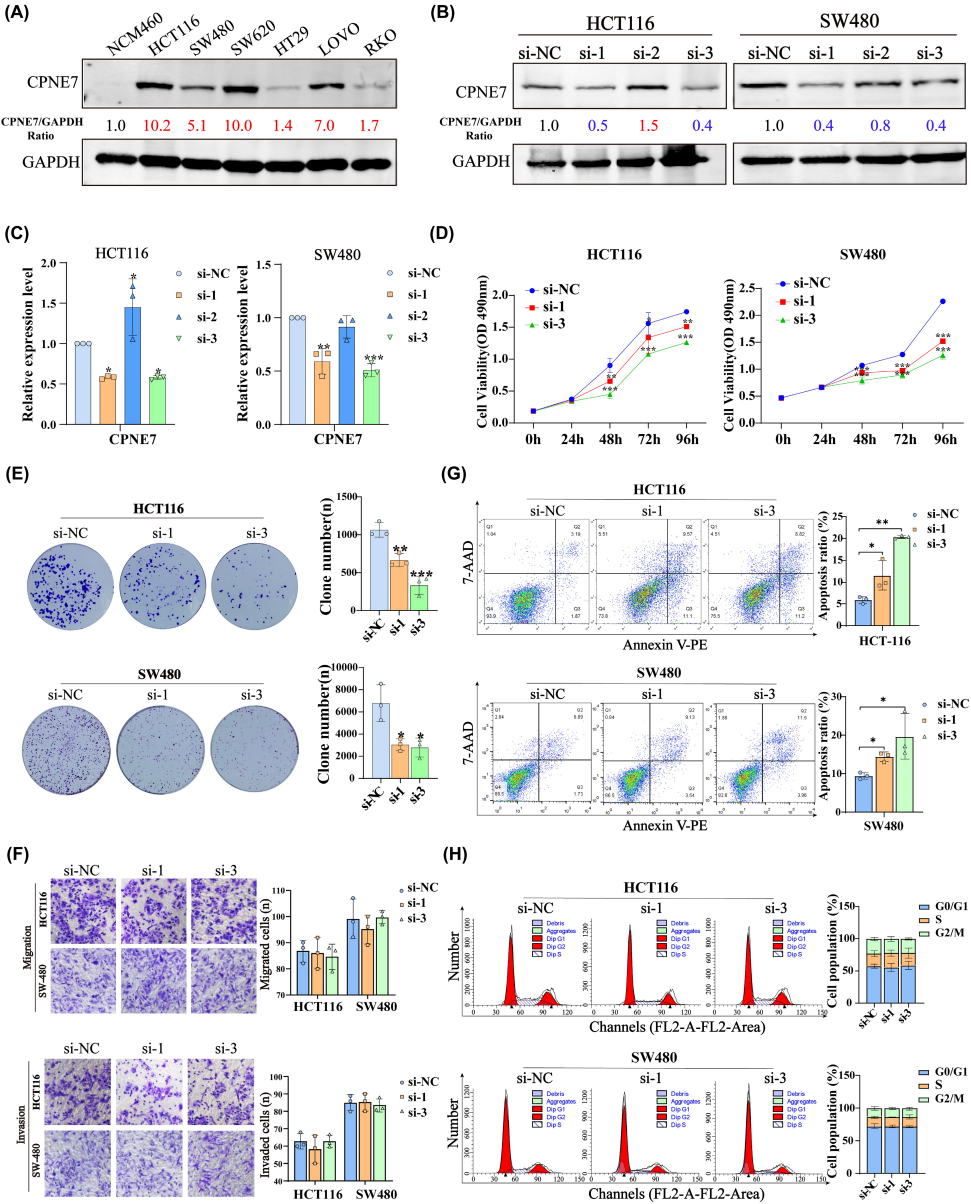
Biological function of CPNE7 in HCT116 and SW480 cell lines. (A) CPNE7 protein expression levels in CRC cell lines. (B, C) Knockdown efficiency of siRNAs for CPNE7. Western blotting and RT‐qPCR were performed to detect CPNE7 expression levels in CPNE7 knockdown (si‐1, si‐2 and si‐3) and negative control (si‐NC) cells. (D) MTS assay. CPNE7 knockdown decreased the viability of HCT116 and SW480 cells. (E) Colony formation assays. CPNE7 knockdown attenuated colony formation in HCT116 and SW480 cells. (F) Transwell migration and invasion assays of CPNE7 knockdown cells. (G) Cell apoptosis assay. Representative flow cytometric images of the si‐NC, si‐1 and si‐3 groups. (H) Cell cycle progression assay of CPNE7 knockdown cells. *p < 0.05, **p < 0.01, ***p < 0.001.

Next, we analysed the expression of CPNE7 in several colorectal cell lines by Western blotting analysis. The results showed that CPNE7 was expressed at low levels in an immortalized intestinal epithelial cell line (NCM460), but was expressed to different degrees in CRC cell lines; in particular, it was highly expressed in the HCT116, SW480, SW620 and LOVO cell lines (Figure 2A). To verify the biological function of CPNE7 in CRC cells, we knocked down the expression of CPNE7 in the HCT116 and SW480 cell lines with three separate siRNAs targeting CPNE7 mRNA. As shown, siRNA‐1 and siRNA‐3 significantly reduced the protein and mRNA levels of CPNE7 (Figure 2B, C). Thus, these two siRNAs were used for follow‐up experiments. Functionally, knockdown of CPNE7 significantly suppressed the proliferation and colony formation abilities of CRC cells (Figure 2D, E), but did not affect the migration or invasion of CRC cells (Figure 2F). Since abnormal cell cycle progression and uncontrolled apoptosis tend to distinctly alter the proliferation and colony formation of tumour cells,^29^, ^30^, ^31^ we also subjected CPNE7‐knockdown CRC cells to cell cycle and apoptosis assays. The depletion of CPNE7 significantly increased cell apoptosis (Figure 2G), but did not significantly change cell cycle progression (Figure 2H). Taken together, these results revealed that CPNE7 truly exerts a pro‐tumour effect in CRC by preventing cell apoptosis.

### 3.3 CPNE7 knockdown represses cellular autophagy and increases 5‐fluorouracil sensitivity in CRC

**FIGURE 3:**
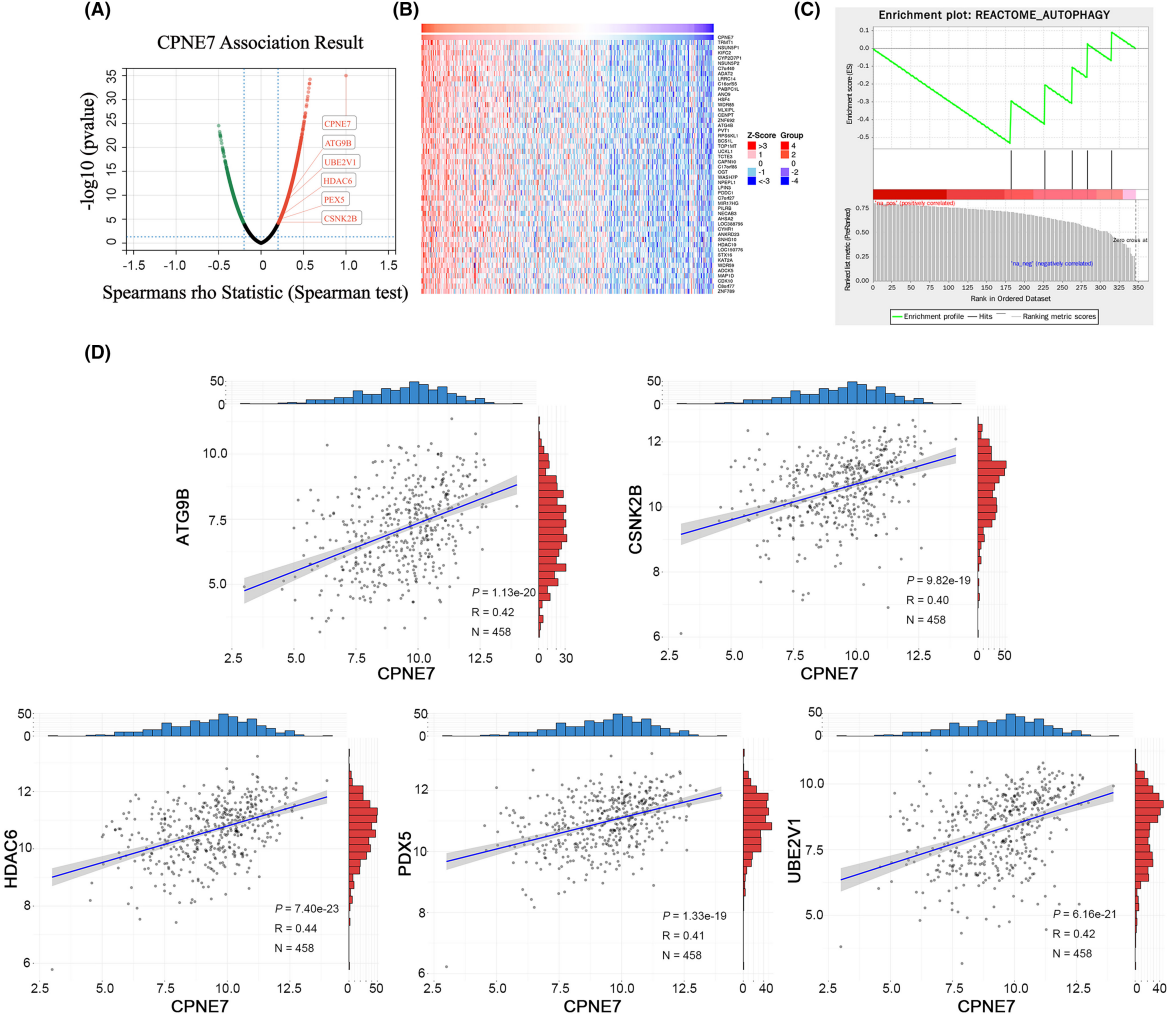
Bioinformatics analysis of the downstream pathway of CPNE7. (A) Hierarchical clustering of the diferentially expressed genes correlated with CPNE7 in CRC patients according to the LinkedOmics database. (B) The top 50 genes positively correlated with CPNE7 are presented in heatmaps. (C) Gene Set Enrichment Analysis (GSEA) was also conducted on the 347 genes. (D) Correlation analysis between the expression of CPNE7 and five genes enriched in the autophagy pathway (ATG9B, CSNK2B, HDAC6, PEX5 and UBE2V1) in CRC patients based on the TCGA database.

**FIGURE 4:**
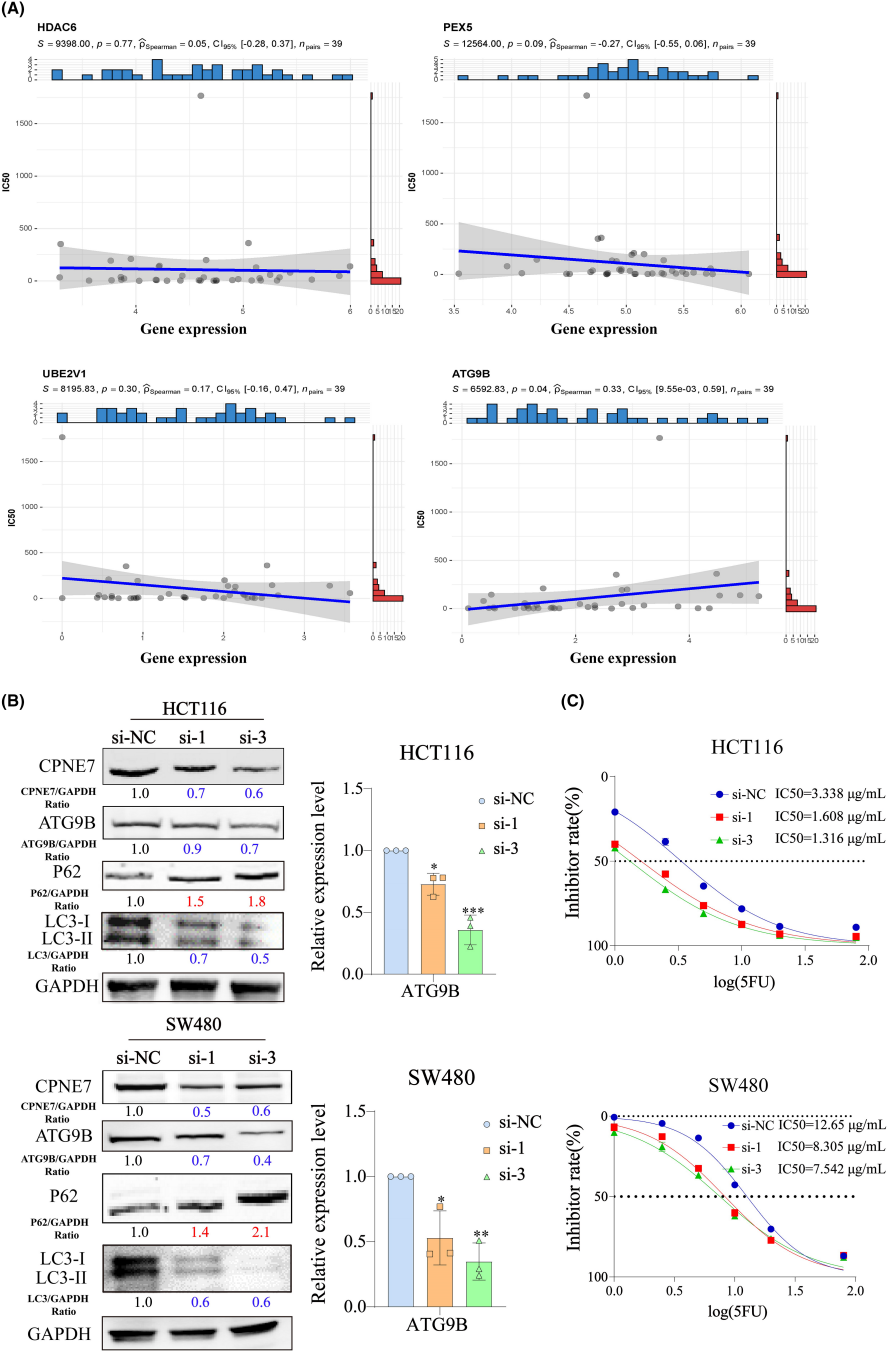
CPNE7 affected the expression of ATG9B and the IC50. (A) Correlation analysis between ATG9B, HDAC6, PEX5 and UBE2V1 expression and the IC50 based on the CCLE and GDSC databases. (B) The mRNA expression level of ATG9B (right) and protein expression level (left) of ATG9B, P62, CPNE7 and LC3‐I/II in both the HCT116 and SW480 cell lines with knockdown of CPNE7. (C) Half‐maximal inhibitory concentration (IC50) analysis. HCT116 (upper), SW480 (lower). *p < 0.05, **p < 0.01, ***p < 0.001.

We demonstrated above that CPNE7 plays a carcinogenic role in CRC. To investigate the downstream genes affected by CPNE7, we performed transcriptome sequencing on CPNE7‐knockdown CRC cells. Through analysis of the transcriptome sequencing data, we found that 5214 genes were downregulated (fold change (FC) < 0.8) (Table S3). Furthermore, we employed LinkedOmics to analyse the RNA‐seq data of 379 CRC patients from the The Cancer Genome Atlas (TCGA) database. We found that 2509 genes correlated with CPNE7 expression (*R*> 0.2) (Figure 3A, Table S4). The heatmap illustrates the expression of the top 50 genes that were positively associated with CPNE7 expression in 379 patients (Figure 3B). To narrow the focus, we intersected the two gene sets (5214 genes and 2509 genes mentioned above) and found that 347 genes were present in both gene sets (Table S5). Then, gene set enrichment analysis (GSEA)^32^, ^33^ was conducted on the these 347 genes, five of which were enriched in autophagy‐related pathways: HDAC6, PEX5, CSNK2B, UBE2V1 and ATG9B (Figure 3C). Next, we downloaded CRC patient data from the TCGA database and conducted correlation analysis of CPNE7 with these five genes. The results showed that CPNE7 was positively correlated with the levels of these five genes, indicating a potential relationship between CPNE7 and these five genes (Figure 3D). To further narrow the range of downstream genes, based on our target pathway (autophagy‐related pathway) and chemotherapy resistance, we conducted a correlation analysis between these five genes and the IC50 value of 5‐fluorouracil. Specifically, we downloaded the gene expression matrix of CRC cell lines from the Cancer Cell Line Encyclopedia (CCLE) database and downloaded the IC50 of 5‐fluorouracil in CRC cell lines from the Genomics of Drug Sensitibity in Cancer (GDSC) database. After integrating the data, we did not observe the expression of CSNK2B in the CCLE database. Therefore, we conducted IC50 correlation analyses for only ATG9B, HDAC6, PEX5 and UBE2V1, and the results showed that only ATG9B was positively correlated with the IC50 (Figure 4A). Previous studies have shown that ATG9B not only regulates cellular autophagy, but is also is correlated with tumour drug resistance,^34^, ^35^ therefore, we selected ATG9B as a potential downstream molecule of CPNE7. We found significant decreases in both the mRNA and protein expression levels of ATG9B in both the CPNE7‐knockdown HCT116 and SW480 cell lines (Figure 4B). In addition, the downregulation of CPNE7 resulted in a significant decrease in LC3‐I/II expression and a significant increase in p62 (an autophagy adaptor protein) expression (Figure 4B). We also analysed the relationship between CPNE7 expression and the sensitivity of CRC cells to 5‐fluorouracil. Compared with that in control group, the sensitivity of CRC cells to 5‐fluorouracil was significantly increased in the group with knockdown of CPNE7 (Figure 4C). In addition, transcription sequencing analysis revealed that other genes related to chemotherapy resistance in CRC, such as ASCL2,^36^ SNHG11^37^ and PVT1,^38^ were significantly downregulated, and these genes are associated with chemotherapy resistance in CRC. In addition, other pathways related to chemotherapy resistance in CRC, such as the MAPK^39^ and RHOA^40^ pathways, were also altered, which proves the impact of CPNE7 on chemotherapy resistance in CRC. Overall, these results indicated that ATG9B expression was regulated by CPNE7 and that knockdown of CPNE7 increased the sensitivity of CRC cells to 5‐fluorouracil.

### 3.4 Knockdown of CPNE7 attenuates rapamycin‐induced autophagy

**FIGURE 5:**
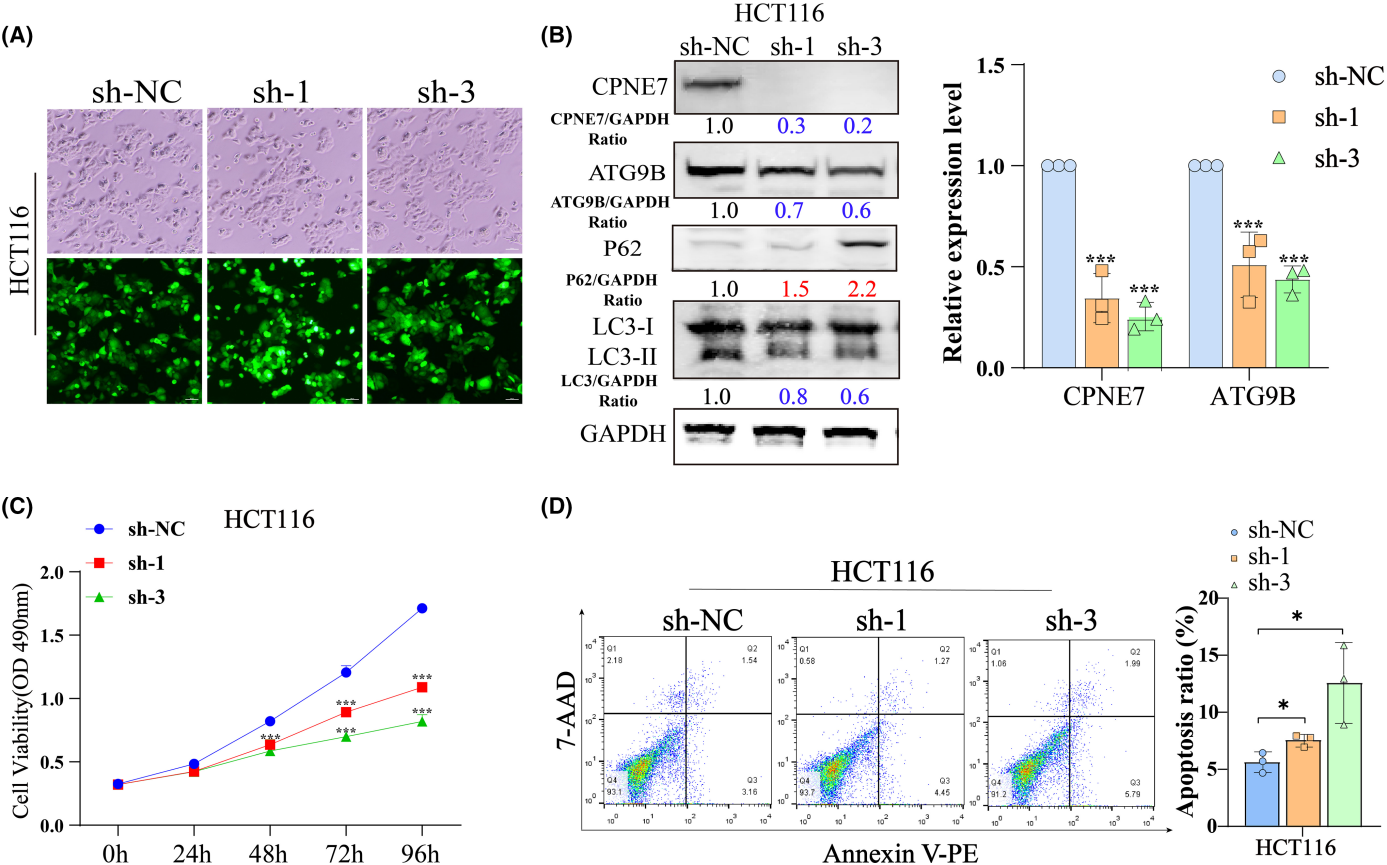
Stable knockdown of CPNE7 affected the malignant behaviours of CRC cells. (A) Stable knockdown of CPNE7 by lentiviral infection in HCT116 cells. (B) ATG9B and LC3‐I/II protein expression levels were significantly downregulated, and p62 was upregulated in CPNE7‐depleted HCT116 cells (left). The mRNA expression of ATG9B and CPNE7 was significantly downregulated in CPNE7‐depleted HCT116 cells (right). (C) MTS assay. The proliferation rate of the HCT116 cell line with stable knockdown of CPNE7 by lentiviral infection was attenuated. (D) Cell apoptosis assay. Apoptosis was increased in CPNE7‐depleted HCT116 cells. *p < 0.05, ***p < 0.001.

**FIGURE 6:**
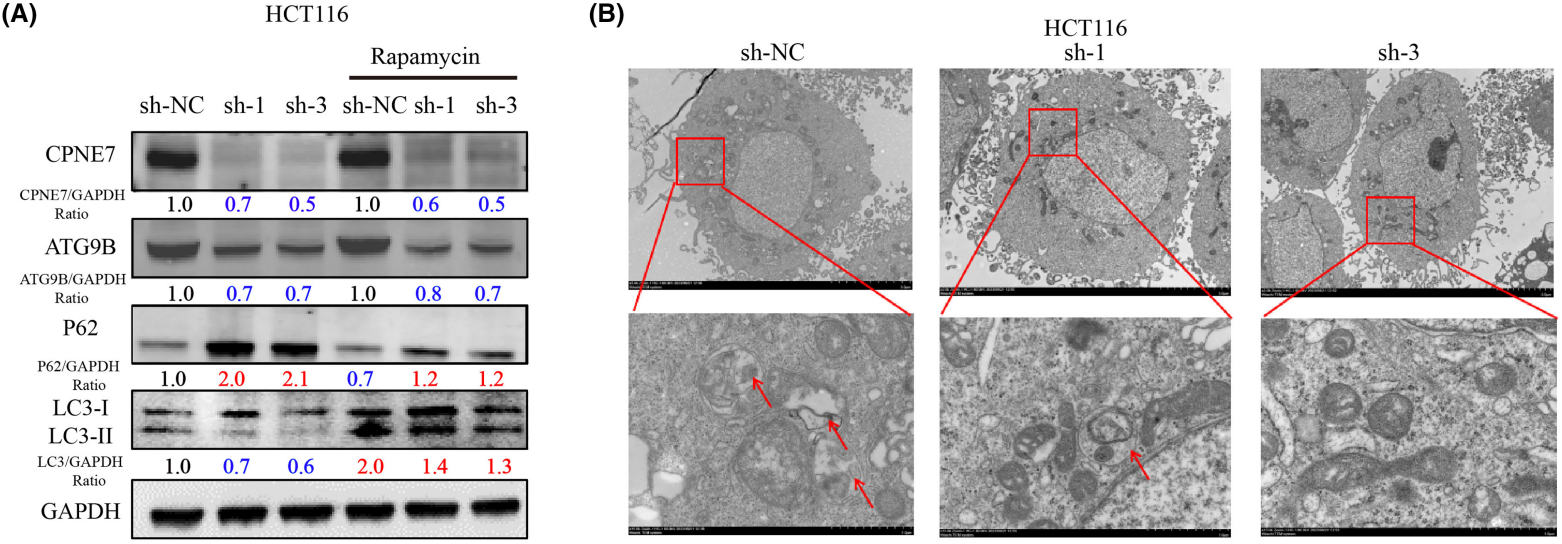
Knockdown of CPNE7 inhibits autophagy. (A) Knockdown of CPNE7 attenuates rapamycin‐induced autophagy. (B) Autophagosomes in the CPNE7 knockdown group and the control group.

We generated HCT116 cells with stable knockdown of CPNE7 via lentiviral infection (Figure 5A). Consistent with the results of transient knockdown experiments, in CPNE7‐knockdown HCT116 cells, the protein expression levels of both ATG9B and LC3‐I/II were significantly decreased, while p62 expression was upregulated (Figure 5B left). The mRNA expression of ATG9B and CPNE7 was significantly downregulated in the CPNE7‐knockdown HCT116 cells (Figure 5B right). The proliferation rate of the cells was markedly attenuated, and the apoptosis rate was markedly increased (Figure 5C, D). However, whether CPNE7 knockdown plays an important role in the autophagy pathway and whether it can attenuate the rapamycin‐induced autophagy pathway are unknown. Our results suggested that the autophagy pathway was activated by the addition of rapamycin, as indicated by an increase in LC3 and a decrease in P62. However, knockdown of CPNE7 attenuated the rapamycin‐induced increase in autophagy (Figure 6A). In addition, the TEM results suggested that the number of autophagosomes was significantly lower in the CPNE7 knockdown group than in the control group (Figure 6B). Thus, our study demonstrated that CPNE7 could be an important target for attenuating or even reversing the autophagic pathway.

### 3.5 Knockdown of CPNE7 suppresses subcutaneous tumour growth and 5‐fluorouracil resistance in vivo

**FIGURE 7:**
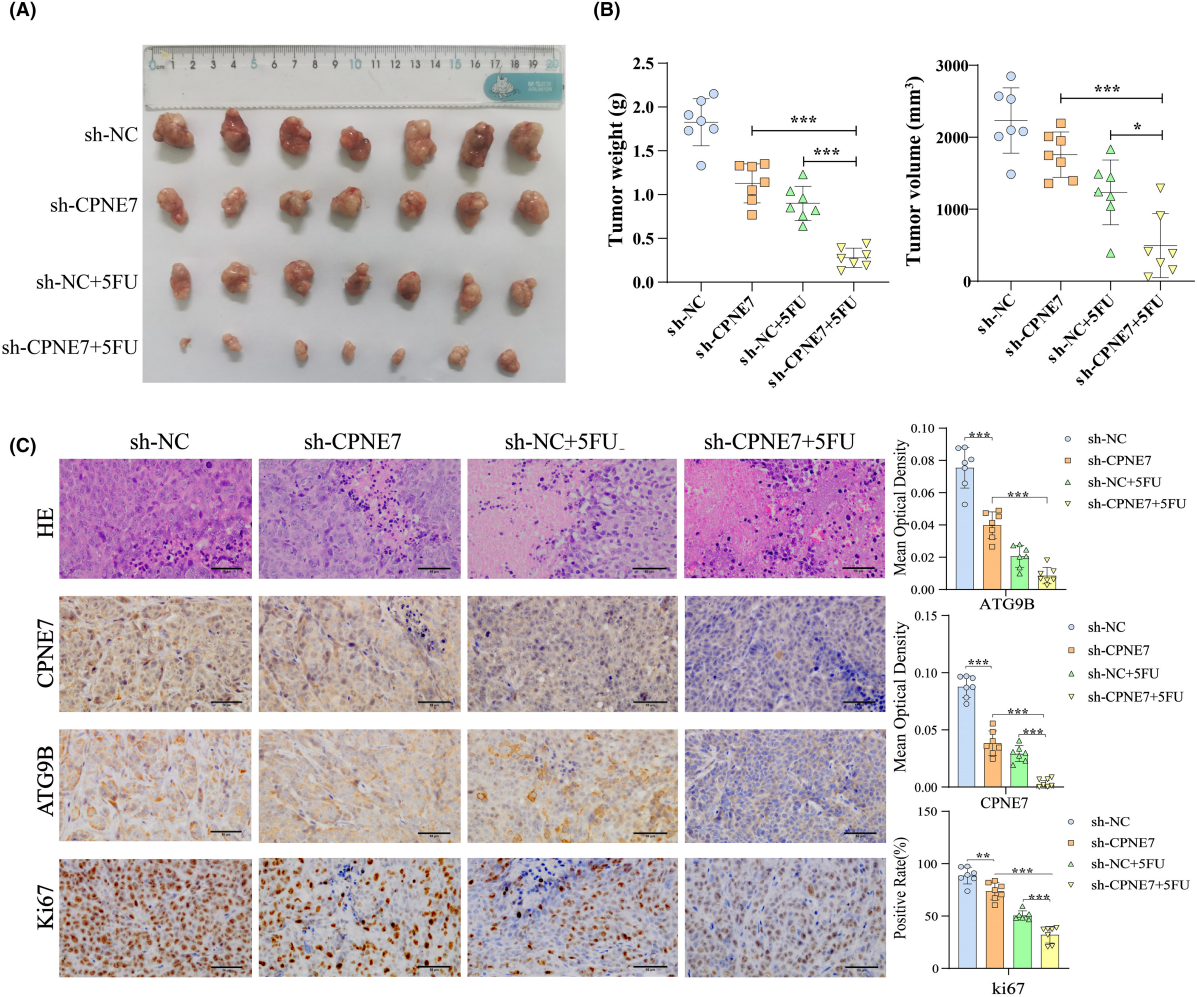
Knockdown of CPNE7 reduces subcutaneous tumour growth and fluorouracil resistance in vivo. (A)The tumours on day 28. (B) Tumour weight (left) and volume (right) were calculated for each group. (C) HE and immunohistochemical staining for ATG9B, CPNE7 and Ki67 in each group (×400). *p < 0.05, **p < 0.01, ***p < 0.001.

In addition, knockdown of CPNE7 significantly inhibited cell proliferation in BALB/c nude mice and enhanced sensitivity to 5‐fluorouracil treatment, as indicated by both tumour volume and weight (Figure 7A, B). Furthermore, we performed HE staining and immunohistochemistry (IHC) on tissues from the different groups of subcutaneously transplanted tumours. The results showed that the most significant decreases in ATG9B and Ki67 expression occurred in CPNE7 knockdown cells treated with 5‐fluorouracil, which indicated that CPNE7 depletion could enhance the sensitivity of cells to 5‐fluorouracil (Figure 7C).

## 4 DISCUSSION

CRC is the second leading cause of cancer‐related death, and its incidence and mortality rates in China are increasing annually.^3^, ^4^ Most patients are in the middle‐late stage when they seek medical help,^41^, ^42^, ^43^ which seriously affects their prognosis. Identification of new CRC markers is important for facilitating the early diagnosis and treatment of CRC.^43^ In our study, we first found that CPNE7 was highly expressed at both the RNA and protein levels in CRC tissues, revealing its potential for use as a biomarker. We also demonstrated that CPNE7 affects cell proliferation and apoptosis in CRC cells, suggesting that CPNE7 could be a therapeutic target. Repurposing of old drugs may be a very important direction. Many drugs, such as gossypol‐acetic acid (GAA) and demethylzeylasteral,^44^, ^45^ can affect the proliferation and chemosensitivity of CRC cells. The prediction of potential targeted therapeutic drugs could also help us narrow down the list of candidate drugs.^46^ In future studies, we will further search for specific inhibitors of CPNE7 that could be used in the clinic. The method used by Zhou et al.^47^ to search for specific inhibitors has provided us with an idea.

We also conducted transcriptome sequencing and found that CPNE7 can affect autophagy‐related signalling pathways. Autophagy is closely related to cell proliferation and chemoresistance.^48^, ^49^ 5‐Fluorouracil is one of the main chemical drugs used for CRC treatment, but the recurrence rate after treatment is greater than 30%, mainly due to the influence of chemical resistance.^50^, ^51^, ^52^ Therefore, the resistance of CRC to chemotherapy is a research focus. In our study, CPNE7 was confirmed to affect autophagy‐related pathway genes, related to chemotherapy resistance. Therefore, we speculated that CPNE7 may also affect the chemosensitivity of CRC cells. A subsequent study showed that CPNE7 could affect the IC50 of 5‐fluorouracil in CRC cells by regulating autophagy, indicating the effect of CPNE7 on chemotherapeutic resistance. We confirmed the effect of CPNE7 on CRC proliferation and chemosensitivity in vitro and obtained consistent results in vivo.

Autophagy plays a critical role in CRC development and chemoresistance.^45^ Targeting autophagy pathways or autophagy‐related molecules to inhibit autophagy in CRC cells has potential therapeutic effects.^46^, ^47^ In our study, after demonstrating that CPNE7 affects autophagy and consequently chemosensitivity in CRC, we further demonstrated the important role of CPNE7 in the autophagy pathway and demonstrated that knockdown of CPNE7 could attenuate or even reverse autophagy. These findings demonstrated the critical role of CPNE7 in the autophagy pathway in CRC and predicted that CPNE7 could be used as a potential therapeutic target that can inhibit the autophagy pathway. This study provides a theoretical basis for the future exploration of drugs targeting CPNE7.

In addition, after knocking down CPNE7, we also found changes in resistance‐related genes, such as ASCL2, SNHG11, PVT1 and ATG9B, as well as resistance‐related pathways, such as MAPK, RHOA and autophagy pathways, further demonstrating the impact of CPNE7 on chemotherapy resistance in CRC and the importance of our study.

### 4.1 Limitations

The samples were obtained from patients in the North China region, and our research was a single‐centre study. In the future, we will expand the sample size and conduct multicentre studies in multiple regions to further clarify the potential of CPNE7 as a biomarker. Moreover, we validated the potential of CPNE7 as a biomarker and therapeutic target, but our study lacked relevant research on the specificity and sensitivity of CPNE7 as a diagnostic marker. In the future, we will collect patient blood and extract the CPNE7 protein or RNA to verify the specificity and sensitivity of using the CPNE7 as a diagnostic marker. In addition, this study did not address the potential heterogeneity of CRC, which may affect the applicability of the CPNE7 signature in different patient subgroups and represents a direction for future research.

Briefly, we demonstrated that CPNE7/ATG9B affects autophagy in CRC cells and thus affects chemotherapeutic resistance. We first investigated the effect of CPNE7 on the expression of ATG9B, which is an autophagy‐related gene that can affect chemotherapeutic resistance. Here, CPNE7 silencing suppressed ATG9B expression and enhanced chemosensitivity. The CPNE7‐induced decrease of ATG9B expression provided us with a strategy for enhancing chemosensitivity. Moreover, CPNE7 silencing inhibited the proliferation and autophagy of CRC cells, indicating the potential of CPNE7 as a therapeutic target. This study provides a therapeutic strategy for patients with high expression of CPNE7 and ATG9B and lays a theoretical foundation for the development and clinical application of antitumour drugs.

## AUTHOR CONTRIBUTIONS

**Weile Xu:** Conceptualization (equal); data curation (equal); investigation (equal); methodology (equal); writing – original draft (equal); writing – review and editing (equal). **Yujie Tang:** Investigation (equal); visualization (equal). **Yang Yang:** Data curation (equal); formal analysis (equal). **Changjing Wang:** Investigation (equal); software (equal). **Chen Liu:** Investigation (equal); validation (equal). **Jianqing Zhang:** Investigation (equal); validation (equal). **Lianmei Zhao:** Methodology (equal); project administration (equal); resources (equal); supervision (equal). **Guiying Wang:** Conceptualization (equal); funding acquisition (equal); investigation (equal); methodology (equal); resources (equal); supervision (equal); writing – original draft (equal); writing – review and editing (equal).

## FUNDING INFORMATION

This work was supported by the National Natural Science Foundation of China (82272909).

## CONFLICT OF INTEREST STATEMENT

The authors declare no conflict of interest.

## Supporting information


Table S1.

Table S2.

Table S3.

Table S4.

Table S5.

## Data Availability

The data that support the findings of our study are available from the corresponding author upon reasonable request.
